# Metabolic changes of glycerophospholipids during the reparative phase after myocardial infarction injury

**DOI:** 10.3389/fcvm.2023.1122571

**Published:** 2023-06-13

**Authors:** Jin Wang, Xinyi Yu, Tingyu Wang, Wenbin Cai, Tong Hua, Jinjie Duan, Xu Zhang, Yi Zhu, Liu Yao

**Affiliations:** The Province and Ministry Co-sponsored Collaborative Innovation Center for Medical Epigenetics, Department of Physiology and Pathophysiology, Tianjin Medical University, Tianjin, China

**Keywords:** myocardial infarction, metabonomics, glycerophospholipids, phosphatidylserine, phosphatidylserine synthase 1

## Abstract

**Introduction:**

Myocardial infarction (MI) is a fatal manifestation of coronary heart disease, and its underlying mechanism is still largely unknown. Lipid levels and composition alterations predict the risk of MI complications. Glycerophospholipids (GPLs) are important bioactive lipids and play a crucial role in the development of cardiovascular diseases. However, the metabolic changes in the GPLs profile during post-MI injury remain unknown.

**Methods:**

In the current study, we constructed a classic MI model by ligating the left anterior descending branch and assessed the alterations in both plasma and myocardial GPLs profiles during the reparative phase post-MI by liquid chromatography–tandem mass spectrometry analysis.

**Results:**

We found that myocardial GPLs, but not plasma GPLs, were markedly changed after MI injury. Importantly, MI injury is associated with decreased phosphatidylserine (PS) levels. Consistently, the expression of phosphatidylserine synthase 1 (PSS1), which catalyzes the formation of PS from its substrate phosphatidylcholine, was significantly reduced in heart tissues after MI injury. Furthermore, oxygen-glucose deprivation (OGD) inhibited PSS1 expression and reduced PS levels in primary neonatal rat cardiomyocytes, while overexpression of PSS1 restored the inhibition of PSS1 and the reduction in PS levels caused by OGD. Moreover, overexpression of PSS1 abrogated, whereas knockdown of PSS1 aggravated, OGD-induced cardiomyocyte apoptosis.

**Conclusions:**

Our findings revealed that GPLs metabolism was involved in the reparative phase post-MI, and cardiac decreased PS levels, resulting from inhibition of PSS1, are important contributor to the reparative phase post-MI. PSS1 overexpression represents a promising therapeutic strategy to attenuate MI injury.

## Introduction

Myocardial infarction (MI), commonly known as a heart attack, is an acute and fatal cardiovascular disease caused by a sharp decrease or cessation of blood supply to the heart muscle, leading to heart damage (1). It is the leading cause of morbidity and mortality worldwide. Diabetes, hypertension, cholesterol and smoking are important risk factors of MI (2), but the pathogenesis of MI remains poorly understood.

Glycerophospholipids (GPLs) or phosphoglycerides, glycerol-based phospholipids, are the main components of the cell membrane and important bioactive lipids. Phosphatidic acid and a substituent group (X) linked to phosphate formed the basic structure of GPLs. According to the different substituent groups linked to phosphate, GPLs can be divided into several classes, including phosphatidylcholine (PC), phosphatidylserine (PS), phosphatidylethanolamine (PE), phosphatidylglycerol (PG) and phosphatidylinositol (PI). In addition, a series of lysophospholipid (LysoPL) metabolized by phospholipases from GPLs to produce lysophosphatidylcholine (LysoPC), lysophosphatidylserine (LysoPS), lysophosphatidylethanolamine (LysoPE), lysophosphatidylglycerol (LysoPG), and lysophosphatidylinositol (LysoPI), are also referred to as GPLs. Previous reports showed that cardiac GPLs have a close relationship with MI (3, 4). In a rat MI model, lysoPC 16:0, lysoPC18:0, lysoPC18:2, lysoPC18:1, lysoPC20:4, and lysoPE18:0 in heart tissues were reported to be increased (3). In the H9c2 rat cardiomyocyte cell line, multiple lipid variations, including PC34:1, PC36:2, PE34:1, PS36:1, PI36:2, PI38:3, and PI38:5, were observed (4). Although GPLs members have been reported to be involved post-MI, the underlying mechanism of GPLs-associated post-MI is unclear.

Cardiac repair after MI is a biphasic wound-healing response that involves an initial inflammatory phase (post-MI days 0–4) and a resolution phase (post-MI days 4–14) (5, 6). In the initial inflammatory phase, infiltrating neutrophils, monocytes and proinflammatory macrophages in the heart serve to clear damaged cells and matrix debris (5, 6). During the resolution phase (post-MI days 4–14), activation of reparative cells relieves inflammation and promotes scar formation and neovascularization (5, 6). PS, one class of GPLs, maintains localization on the inner surface of the cell membrane (7). Externalization of PS represents PS transfer from the inner leaflet to the outer leaflet promoting cell apoptosis, phagocytosis, and ultimately degradation by phagocytes; simultaneously, it causes an “inflammo-suppression” state by producing anti-inflammatory factors such as IL-10 and TGF-β (8). Reports have indicated that oral PS markedly reduced MI size and prevented adverse left ventricular remodeling in a mouse model of acute MI (9). Targeting of PS-presenting liposomes to infarct macrophages after injection via the femoral vein promoted angiogenesis and the preservation of small scars and prevented ventricular dilatation and remodeling (10). Therefore, PS plays a pivotal role in cardiac repair after MI.

In mammalian cells, several active enzymes, including phosphatidylserine synthase 1 (PSS1), phosphatidylserine synthase 2 (PSS2), PE N-methyltransferase (PEMT), and PS decarboxylase (PISD), dominantly regulate PS metabolism (11, 12). PSS1 and PSS2 are localized in mitochondrial-associated membranes, a specific region in the endoplasmic reticulum (ER) that is reversibly tethered to mitochondria (11, 12), directly involved in PS dynamic synthesis, while PEMT is localized in mitochondrial-associated membranes and the ER, and PISD is found in mitochondria (11, 12), indirectly involved in PS dynamic synthesis and directly promoting PS transformation. A recent study noted that PSS1 deficiency led to low PS levels, which destroyed normal LDL cholesterol transportation and induced toxic cholesterol accumulation (13). In addition, inhibiting PS synthesis caused by compensatory reduced expression of PSS1 and PSS2 in mitochondrial protein mitofusin 2-deficient mouse livers promoted ER stress and led to the development of a nonalcoholic steatohepatitis-like phenotype and liver cancer (14).

In this study, we demonstrated that the cardiac metabolic profile of GPLs, but not plasma, exhibited significant variation at the reparative phase after MI surgery in mice. PS primarily participated in this process, and myocardial PSS1 was identified as a key regulator of PS production. Our results may provide a therapeutic target for MI.

## Materials and methods

### Animal model

Eight-week-old male C57BL/6 mice were procured from Beijing Vital River Laboratory Animal Technology (Beijing, China). MI was induced through permanent ligation of the left anterior descending branch of the coronary artery (LAD ligation) as described previously (15). Briefly, mice were anesthetized with 3% isoflurane 1 L/min of 100% O_2_ and fixed on a 37°C constant temperature plate during the operation. After the mice were anesthetized, an approximately 2 cm incision was cut at the position where the heart beats the most. Then, a 6-0 medical surgical suture was used to quickly tie a surgical knot on the left anterior descending coronary artery, and the sham mice were only opened in the chest and pericardium without LAD ligation. Finally, the mouse thoracic cavity was closed and sutured. The animal study was reviewed and approved by the Committee of Tianjin Medical University on Animal Experimentation.

### Echocardiography

We monitored mouse cardiac function via echocardiography. Briefly, the heart mode of the mouse heart ultrasound was detected by a 30 MHz probe. The left chest of each mouse was exposed, and a clear long-axis cross-section of the heart in B mode was traced and recorded. Three consecutive short-axis cardiac cycle parameters were used to calculate the dimensions of the systolic and diastolic myocardium according to the VisualSonics standard measurements.

### TTC-Evans blue double staining and H&E staining

The infarct area of heart tissues after MI was stained with 2,3,5-triphenyltetrazolium chloride (TTC)-Evans blue and hematoxylin and eosin (H&E) staining. Briefly, for TTC-Evans staining, mice were anesthetized, and their hearts were injected with 1 ml of 0.5% Evans blue via the thoracic aorta after occlusion of the left anterior descending branch. Mouse hearts were removed, washed with phosphate buffered saline and frozen at −20°C for 30 min. Heart sections (1 mm) from the ligation to the apex were obtained and placed in 1% TTC at 37°C for 15–20 min for staining. The remote area was stained blue, the area at risk was stained red, and the infarct area was visualized by unstained white. For H&E staining, heart tissues were fixed in 10% neutral buffered formalin overnight and then embedded in paraffin wax. Sequential 5 µm paraffin-embedded sections were prepared and stained with hematoxylin and eosin to evaluate morphological changes.

### Immunofluorescence staining

Mouse hearts were fixed and embedded in optimal cutting temperature compound before sectioning. Frozen heart sections were first incubated with blocking buffer (phosphate buffered saline containing 5% goat serum and 0.3% Triton X-100) at 25°C for 1 h and then incubated with primary antibody at 4°C overnight. Sections were washed with phosphate buffered saline and then incubated with secondary antibodies for 1 h at room temperature. The following antibodies were used: anti-PSS1 (1:100) and anti-cTnl (1:100). Fluoroshield mounting medium with DAPI was used to cover slides, and images were captured using a Zeiss confocal laser-scanning microscope. Images of fluorescence intensity were quantified with ImageJ software.

### TUNEL staining

For terminal deoxynucleotidyl transferase-mediated dUTP *in situ* nick-end labeling (TUNEL) staining, a TUNEL FITC apoptosis detection kit (Abbkine) was used to evaluate the cardiomyocyte apoptosis. Briefly, primary neonatal rat cardiomyocytes were fixed with 4% paraformaldehyde solution for 20 min, permeabilized with 0.5% Triton X-100 for 20 min and blocked with 5% bovine serum albumin in phosphate buffered saline-Tween for 1 h. DAPI was used for nuclear counterstaining, and the number of TUNEL+ nuclei was counted. Images were captured by fluorescence microscopy (Zeiss, Germany).

### Metabolomics analysis

Metabolomic analysis involved liquid chromatography–tandem mass spectrometry (LC–MS/MS) of metabolites as described previously (16, 17). The methyl-tert-butyl ether (MTBE)-based method was selected to detect lipid levels in mouse plasma and hearts. Briefly, 200 µl plasma or 50 mg heart tissue was spiked with an internal standard mixture. Then, 400 µl 75% methanol was added and mixed. Subsequently, 1 ml MTBE was supplemented and incubated with the mixture for 1 h. Then, 250 µl of sterile water was added to induce phase separation. Finally, the upper organic phase was transferred to a new tube, and the water phase was extracted again. The organic phase was combined and then evaporated to dryness after centrifugation for 10 min at 12,000×*g*.

Target profiling of GPLs metabolites was performed using a 5500 QTRAP hybrid triple quadruple linear ion-trap mass spectrometer (AB Sciex, Foster City, CA, USA) equipped with a turbo ion-spray electrospray ionization source. For data analysis, we used Metaboanalyst 3.0 (http://www.metaboanalyst.ca). Missing values were imputed with half of the minimum positive value, and data were log-transformed and autoscaled before analysis.

### Isolation and culture of primary neonatal rat cardiomyocytes, adenoviral infection and transient transfection

Primary neonatal rat cardiomyocytes were isolated and cultured as described previously (18). Briefly, cardiomyocytes were isolated from 1- to 2-day-old Sprague‒Dawley rats with Hank's balanced salt solution containing 0.1% trypsin and 0.05% collagenase type II. After 2 h of differential adherence, the isolated cells were cultured in Dulbecco's Modified Eagle Medium containing 10% fetal bovine serum for 48 h. Oxygen–glucose deprivation (OGD) is a well-recognized model of ischemic injury in cardiomyocytes. Primary neonatal rat cardiomyocytes were infected with adenoviral PSS1(Ad-PSS1) for 36 h and then cultured in 1,640 medium deprived of glucose in a VIOS 160i incubator (Thermo Fisher Scientific, Madison, WI, USA) filled with 94% N_2_, 5% CO_2_ and 1% O_2_ for 12 h. Finally, the cells were lysed and collected for experiments.

For gene knockdown experiments, primary neonatal rat cardiomyocytes were transfected with siRNA-negative control (siNC) or PSS1 (siPSS1) for 36 h by use of Lipofectamine 2000. Then cells were lysed and collected for experiments.

### Total RNA isolation and quantitative PCR (qPCR)

Total RNA from mouse hearts or rat cardiomyocytes was isolated using RNA extraction kits (TransGen Biotech). RNA samples were reverse transcribed, and cDNA samples were used as templates for qPCR. qPCR was performed with Brilliant II SYBR Green qPCR Master Mix (Stratagene, CA, United States) and the StepOnePlus qPCR System (Applied Biosystems, Waltham, MA). The levels of each mRNA were normalized to that of 18S and the level in the normal control. Sequences of primers as following: 18S (F:5′-GGAAGGGCACCACCAGGAGT-3′, R:5′-TGCAGCCCCGGACATCTAAG-3′) mouse PSS1 (F:5′-CCGTAGTTATGGGCTTTGC-3′, R:5′-GCTTGATCTTCCCTGTGGT-3′); mouse PSS2 (F:5′-AGTCTCCGCTGCTCAAGG-3′, R:5′-CTCCAAAACATAAGAAAACCAA-3′); mouse PEMT (F:5′-TGGTAGCGAGATGGGAGCA-3′, 5′-CAGTGGGAGCGGAGGATGT-3′); mouse PISD (F:5′-GACTGGACCATCTCACATC-3′, 5′-AGTTAGGACTACACGCTCA-3′); Rat PSS1 (F:5′-ACTACGCCTACCTCACG-3′, 5′-AAAGAAGCCAAAGCACC-3′).

### Western blotting

Total protein was extracted and separated by 10% or 12.5% SDS‒PAGE and then transferred to an NC membrane, which was immunostained with primary antibodies for PSS1 at 4°C overnight. To control for unwanted sources of variation, we normalized the relative expression of target proteins in various groups to that of GAPDH.

### Statistical analysis

Data are presented as the mean ± standard error of the mean (SEM). Differences between groups were analyzed by unpaired *t*-test, two-way ANOVA with Bonferroni's posttest for comparisons of more than two groups. Statistical significance was set at *P *< 0.05. Statistical analysis involved the use of GraphPad Prism software (version 5.01; GraphPad Software Inc., San Diego, CA).

## Results

### Analysis of the plasma GPLs profile after MI injury

**Figure 1:**
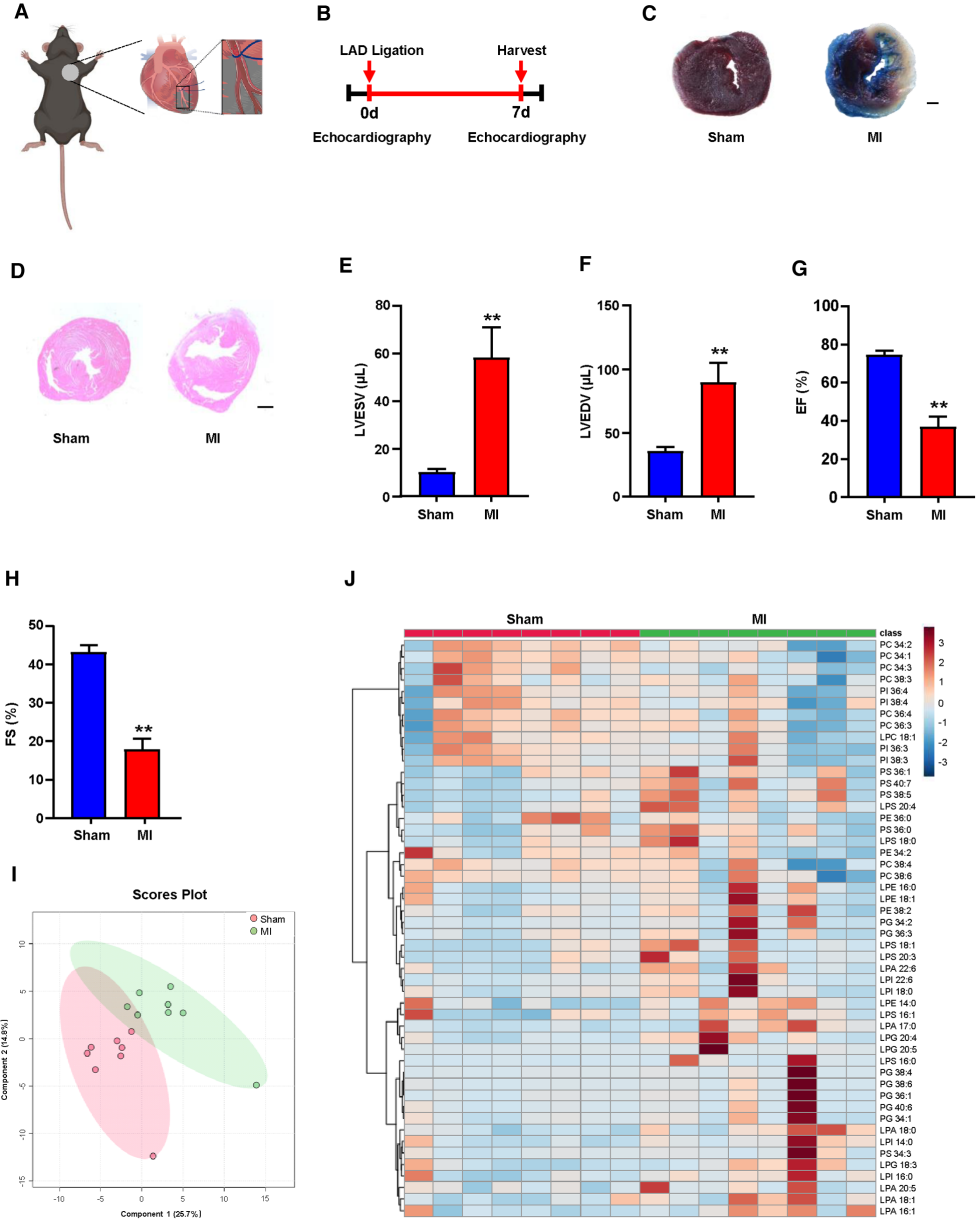
Plasma glycerophospholipids (GPLs) profile in sham and myocardial infarction (MI) mice. (**A**) A model diagram of MI in mice. (**B**) Timeline of the MI experiment. (**C–H**) MI surgery was performed in C57BL/6 mice, and related variables were assessed on day 7 post-MI. (**C**) 2,3,5-Triphenyltetrazolium chloride (TTC)-Evans blue staining and (**D**) hematoxylin and eosin (H&E) staining of left ventricular (LV) tissue sections of sham and MI mice. Scale bar, 500 µm (**C**) and 1 mm (**D**). (**E–H**) Quantification of LV end-diastolic volume (LVEDV), LV end-systolic volume (LVESV), LV ejection fraction (EF), and LV fraction shortening (FS) by echocardiographic analysis. (**I,J**) Liquid chromatography–tandem mass spectrometry (LC—MS/MS) detected GPLs levels in mouse plasma. (**I**) An orthogonal partial least squares discriminant analysis (PLS-DA) model was built based on metabolite measurements. (**J**) Heatmap of GPLs and their associated lysophospholipids levels in the plasma of sham and MI mice. Quantified data are the mean ± SEM of 8 mice in each group. Unpaired 2-tailed *t*-test, **P < 0.01 vs. sham group.

To determine the relevance of the GPLs profile and the reparative phase of MI injury *in vivo*, we performed ligation of the left anterior descending (LAD) of mice to establish an MI model (Figure 1A). Seven days after MI (Figure 1B), we used TTC staining and H&E staining of the left ventricle (LV) to display morphological changes in the heart and employed echocardiography to detect cardiac function. TTC staining and H&E staining of the LV validated obvious cardiac tissue damage on day 7 post-MI (Figures 1C,D). LV systolic function or LV remodeling was assessed by LV end-systolic volume (LVESV), LV end-diastolic volume (LVEDV), ejection fraction (EF) and fractional shortening (FS). LVESV and LVEDV were significantly higher (Figures 1E,F), while EF and FS were markedly lower than sham controls (Figures 1G,H).

Plasma lipid metabolism is commonly associated with the development of MI (19–21). To explore the possible correlation between the GPLs profile and MI, we first used targeted LC‒MS/MS to determine the changes in the GPLs profile in plasma. Based on the measured metabolites, including GPLs and their derived lysophospholipids, orthogonal partial least squares discriminant PLS-DA analysis was used to establish a classification model for the GPLs profile between sham and MI. The 2D score diagram of PLS-DA revealed no separation of GPLs and their associated lysophospholipids between the sham and MI groups (Figure 1I). Furthermore, heatmap analysis of the top 50 metabolites exhibited a comparable the GPLs profile and their associated lysophospholipids between these 2 groups (Figure 1J). These data suggested that plasma GPLs metabolism is not relevant to post-MI.

### Cardiac GPLs profile was significantly altered, and the PS family is an important contributor to MI injury

**Figure 2:**
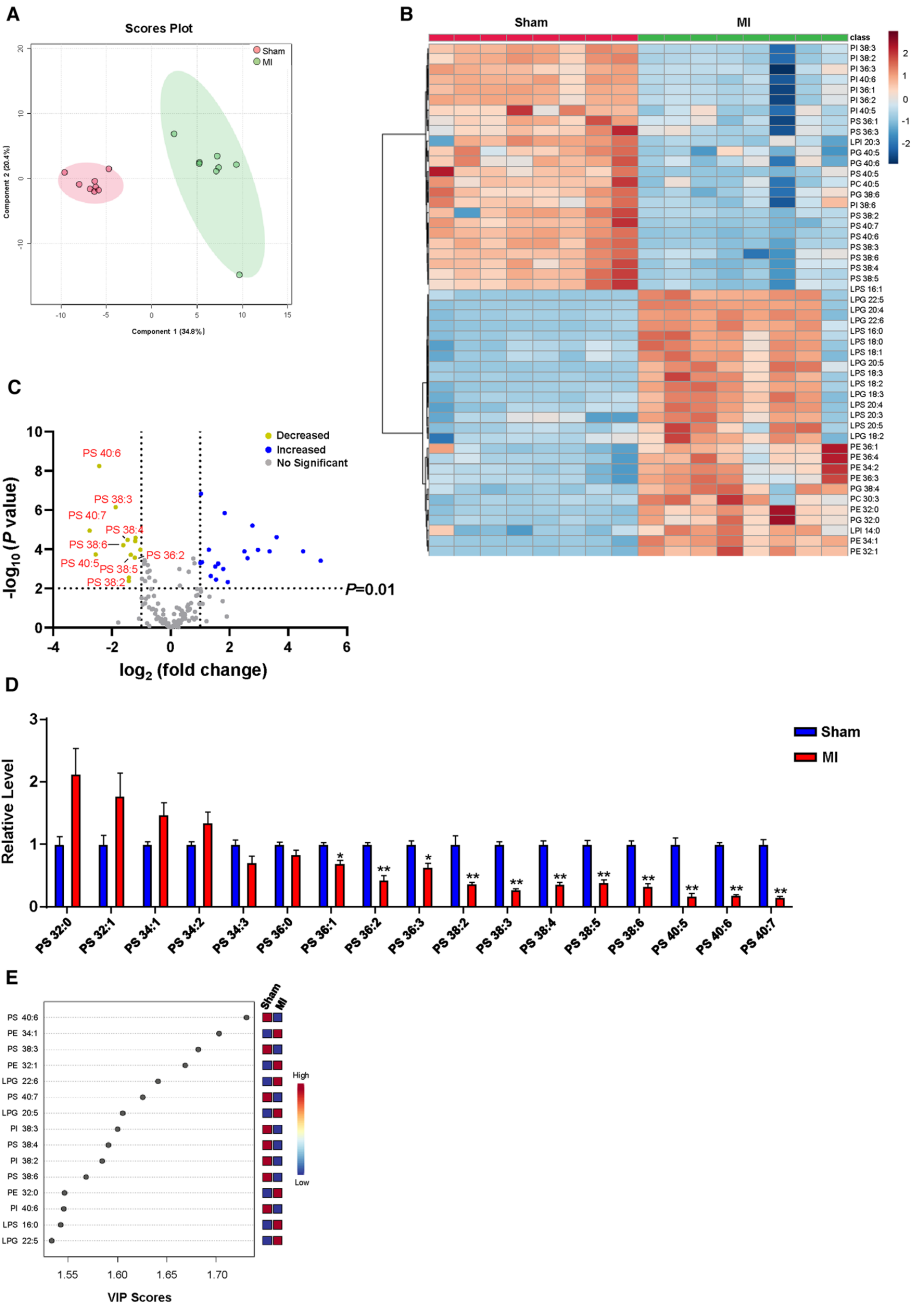
Myocardial GPLs profile of sham and MI mice at day 7 post-MI. (**A**) PLS-DA score plot of the GPLs profile. (**B**) Heatmap of GPLs levels in heart tissues of sham and MI mice. (**C**) Volcano plot showing the fold change in metabolites under false discovery rate (FDR) correction between sham and MI mice hearts. (**D**) Changes of PS levels in cardiac tissues of MI mice. (**E**) Features (variables) of the top 15 most significant metabolites based on VIP scores from PLS-DA. The *x*-axis shows correlation scores, and the *y*-axis shows the metabolites. Quantified data are the mean ± SEM of 8 mice in each group. Two-way ANOVA, *P < 0.05; **P < 0.01 vs. sham group.

Numerous correlation studies have provided a link between cardiac lipid metabolism and post-MI, with a focus on arachidonic acid metabolism (15, 22), omega-3 polyunsaturated fatty acid metabolism (23), and sphingolipid metabolism (24, 25). However, the role of cardiac GPLs metabolism in post-MI remains largely unknown. To identify cardiac GPLs variations that are potentially involved in post-MI, we screened the change in the GPLs profile in heart tissues of MI and sham controls by targeted LC‒MS/MS. PLS-DA analysis showed a marked separation of the GPLs profile and their associated lysophospholipids between the sham and MI groups (Figure 2A). In addition, heatmap analysis of the top 50 metabolites indicated a remarkable difference between the sham and MI groups (Figure 2B). To identify metabolites that are potentially involved in MI injury, we analyzed the changes in GPLs and their associated lysophospholipids by volcano plot in cardiac tissues of the MI and sham groups. Metabolites above the horizontal dashed line have *P* < 0.01 after false discovery rate (FDR) correction. It is evident that there are 13 decreased metabolites in the upper left corner (>2-fold) and 19 increased metabolites in the upper right corner (>2-fold). Of note, among the 13 decreased metabolites, 9 were PS family members (PS 40:6; PS 38:3; PS 40:7; PS 38:4; PS 38:6; PS 40:5; PS 36:2; PS 38:5; PS 38:2) (Figure 2C). Accordingly, statistical analysis of all the detected PS family members in heart tissues confirmed the significant reductions in these 9 PS family members (Figure 2D). Importantly, variable importance for projection (VIP) score analysis identified the top 15 metabolites, which contributed to the great difference between the sham and MI groups. PS (40:6) was the most different metabolite (Figure 2E). These results suggest that reduced PS levels primarily contributed to MI injury.

### PSS1 expression is decreased in cardiac tissues of post-MI mice

**Figure 3:**
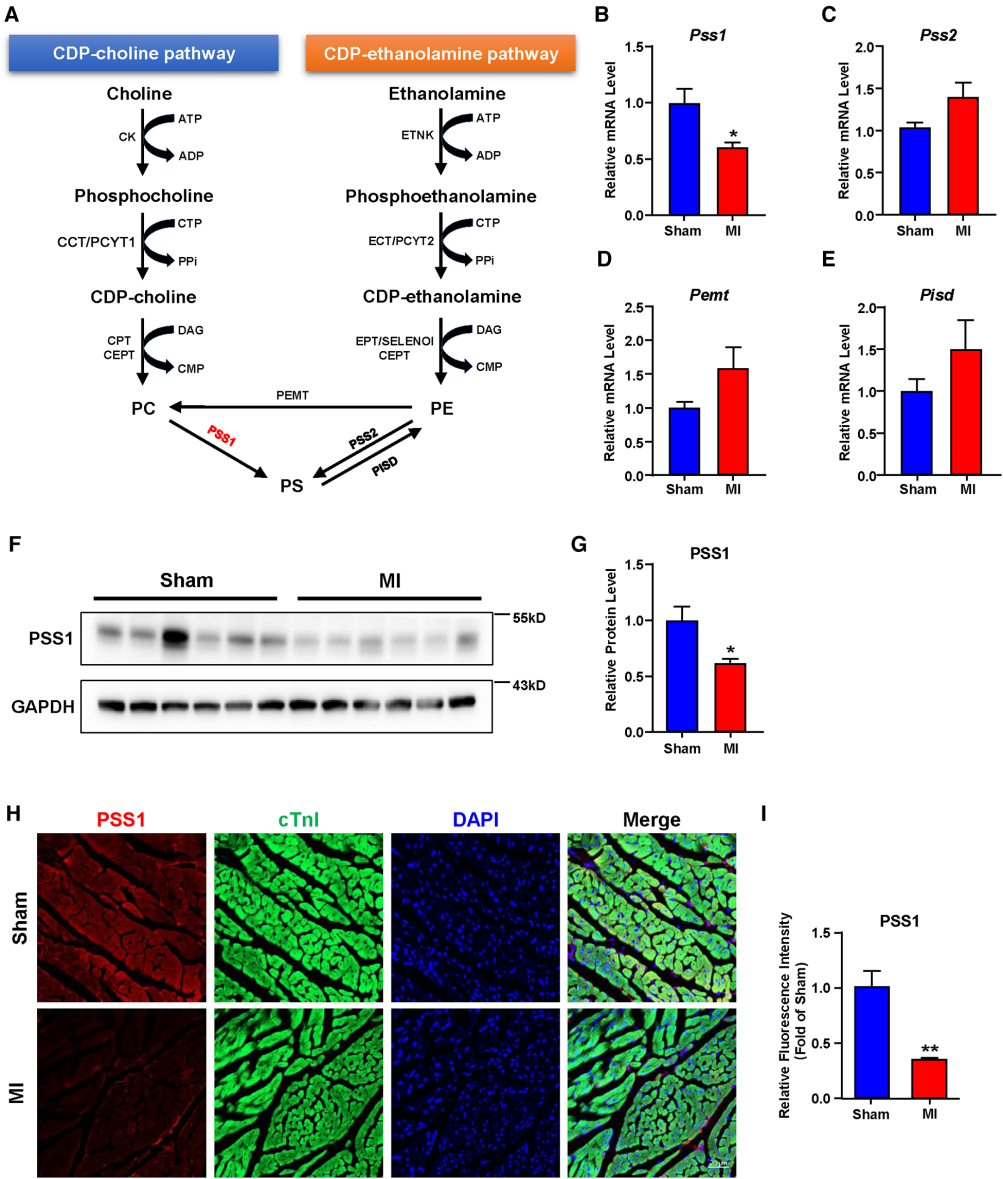
Gene expression related to phosphatidylserine (PS) synthesis and transformation in the hearts of sham and MI mice at day 7 post-MI. (**A**) Metabolic pathways of PS synthesis and transformation. (**B–E**) mRNA levels of genes related to PS synthesis and transformation. (**F–G**) Western blotting analysis (**F**) and quantification (**G**) of the protein level of PSS1 in cardiac tissues of mice. (**H**) Representative immunofluorescence staining images of PSS1 in the myocardium. Scale bar, 20 µm. (**I**) Fluorescence intensity quantification of PSS1 in the myocardium. Quantified data are the mean ± SEM of 5–8 mice in each group. Unpaired 2-tailed *t*-test, *P < 0.05; **P < 0.01 vs. sham group.

In mammalian cells, dynamic synthesis and transformation maintain PS levels. As illustrated in Figure 3A, PS can be synthesized directly from PC and PE or obtained indirectly from PE by conversion to PC. The synthesized PS can be metabolized to PE again. Three major biosynthetic enzymes, PSS1, PSS2 and PEMT, are involved in PS formation. Then, PISD could catalyze PS to PE again. Analysis of these genes involved in PS biosynthesis and transformation showed that the mRNA and protein levels of PSS1 were reduced in cardiac tissues of post-MI mice (Figures 3B,F,G), with no significant alteration in PSS2, PEMT and PISD (Figures 3C–E). Subsequently, the intensity of PSS1 in the border area of cardiac tissue post-MI was weaker than that in the non-infarcted area (Figures 3H,I), which was concomitant with decreased mRNA and protein levels of PSS1. Thus, downregulation of PSS1 in post-MI cardiac tissue inhibited PS synthesis and then promoted heart damage.

### PSS1 is primarily expressed in cardiomyocytes

**Figure 4:**
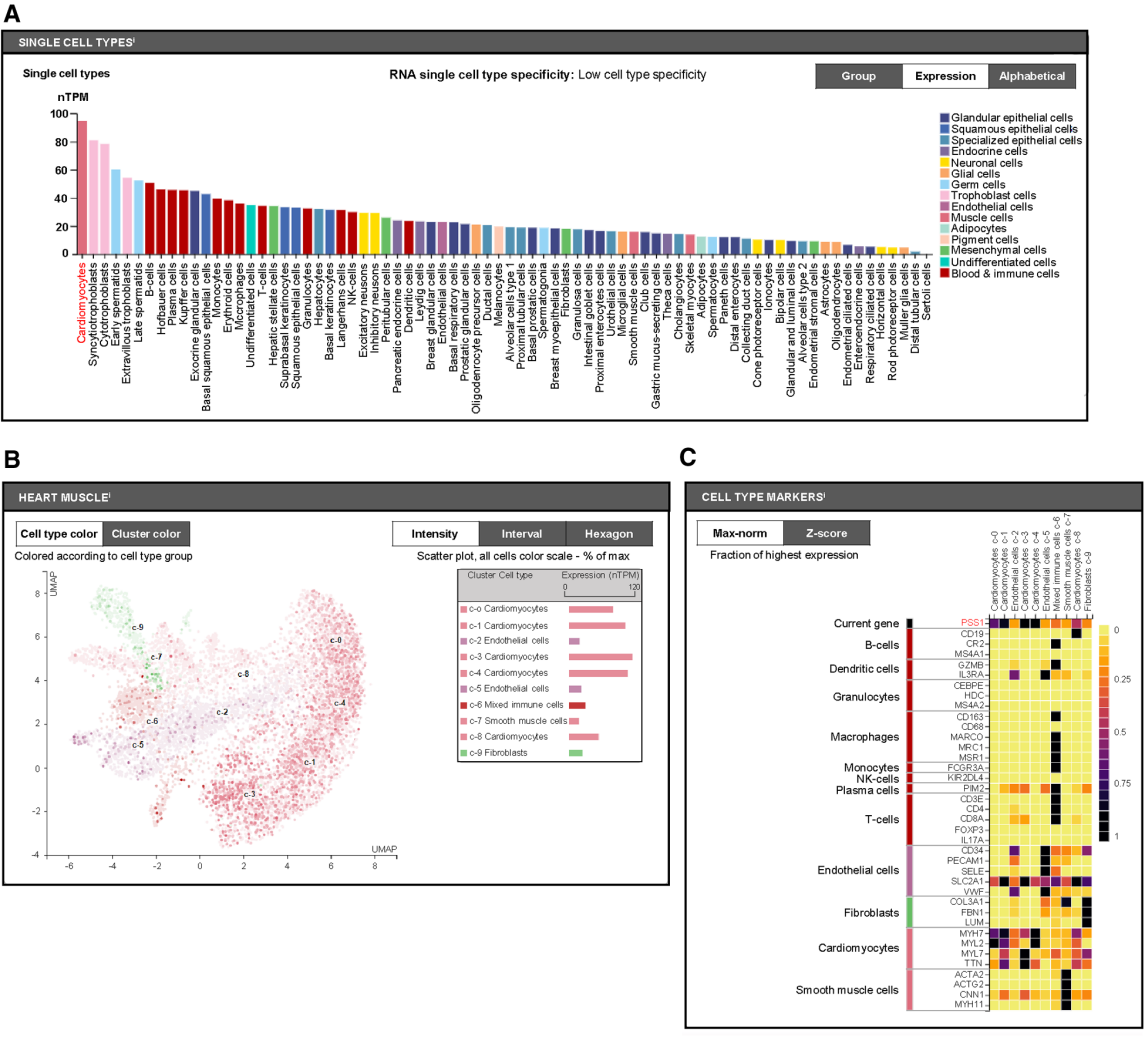
Cell type expression of phosphatidylserine synthase 1 (PSS1). (**A**) Single-cell analysis of PSS1 in different human cell types via the Human Protein Atlas online website (https://www.proteinatlas.org). (**B**) Comparison of PSS1 expression in different cell type clusters of heart muscle. Different colors represent different cell type clusters in heart muscle, with nTPM values reflecting the corresponding PSS1 expression. Normalized gene expression values are reported as nTPM. (**C**) Evaluation of the expression of PSS1 in different cell type clusters of heart muscle by the fraction with the highest expression. The deep purple or even black color represents the highest PSS1 expression fraction; light yellow or orange color represents a relative low PSS1 expression fraction, with specific markers to distinguish cardiac cell types.

Furthermore, we aimed to distinguish which cell types with PSS1 expression in heart tissues are mainly involved in the regulation of PS production and MI injury and remodeling. We used the human protein atlas online website (https://www.proteinatlas.org) to predict PSS1 expression in humans. According to single-cell analysis of PSS1 in different human cell types, we found it shows highest level in cardiomyocytes (Figure 4A). Subsequently, further single-cell analysis of PSS1 expression in different cell type groups of heart muscle also verified its higher expression in cardiomyocytes than in noncardiomyocytes, including mixed immune cells, fibroblasts, endothelial cells, and smooth muscle cells (Figures 4B,C). Hence, cardiomyocyte PSS1 is the most important regulator of PS production and MI injury and remodeling.

### Change of PSS1 expression affected PS levels and OGD-induced cardiomyocytes apoptosis

**Figure 5:**
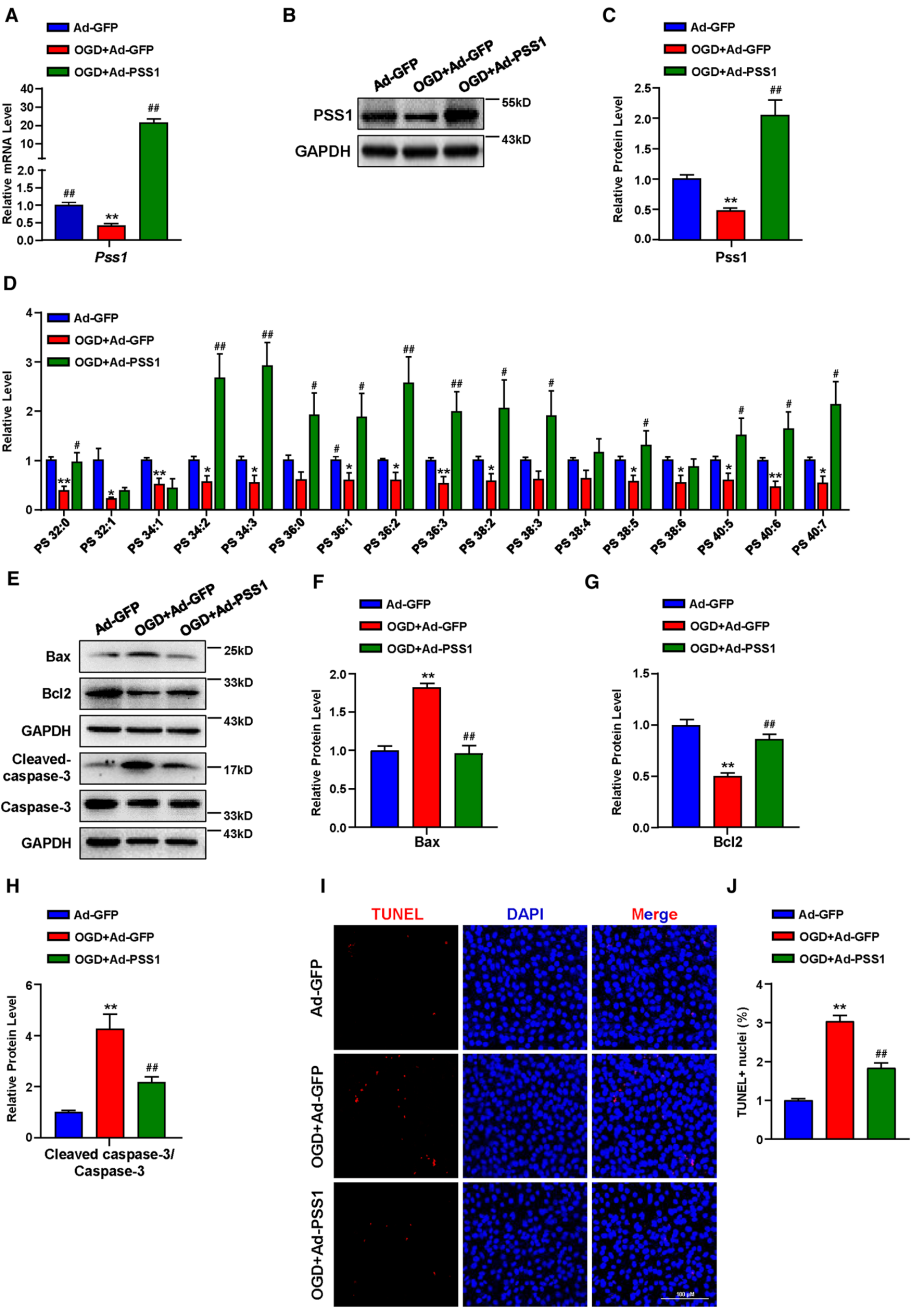
Effects of PSS1 overexpression on cardiomyocyte PS synthesis and apoptosis under oxygen-glucose deprivation (OGD). Cardiomyocytes were infected with adenoviral PSS1 and then treated with OGD. (**A**) mRNA level of *Pss1*. (**B,C**) Western blotting analysis (**B**) and quantification (**C**) of the protein level of PSS1. (**D**) PS levels in cardiomyocytes detected by LC—MS/MS. (**E–H**) Western blotting analysis (**E**) and quantification (**F–H**) of apoptosis-associated protein levels of Bax, Bcl2 and the ratio of cleaved-caspase-3/caspase-3. (**I,J**) Representative images of immunofluorescence staining (**I**) and quantification (**J**) of terminal deoxynucleotidyl transferase dUTP nick end labeling (TUNEL) positive cardiomyocytes. Quantified data are the mean ± SEM of 5 independent experiments. Two-way ANOVA, *P < 0.05, **P < 0.01 vs. Ad-GFP; *^#^P < *0.05, *^##^P < *0.01 vs. OGD + Ad-GFP. Scale bar: 100 µm.

To determine the significance of PSS1 in MI *in vitro*, we evaluated its expression in primary neonatal rat cardiomyocytes under OGD, which mimics ischemia treatments. Both the mRNA and protein levels of PSS1 were significantly downregulated in primary neonatal rat cardiomyocytes after OGD (Figures 5A–C). Evaluation of PS levels by targeted LC‒MS/MS revealed that OGD significantly reduced most PS levels in cardiomyocytes (Figure 5D). However, overexpression of PSS1 by adenovirus infection of cardiomyocytes reversed the inhibition of PSS1 and the reduction in PS levels caused by OGD (Figures 5A–D).

**Figure 6:**
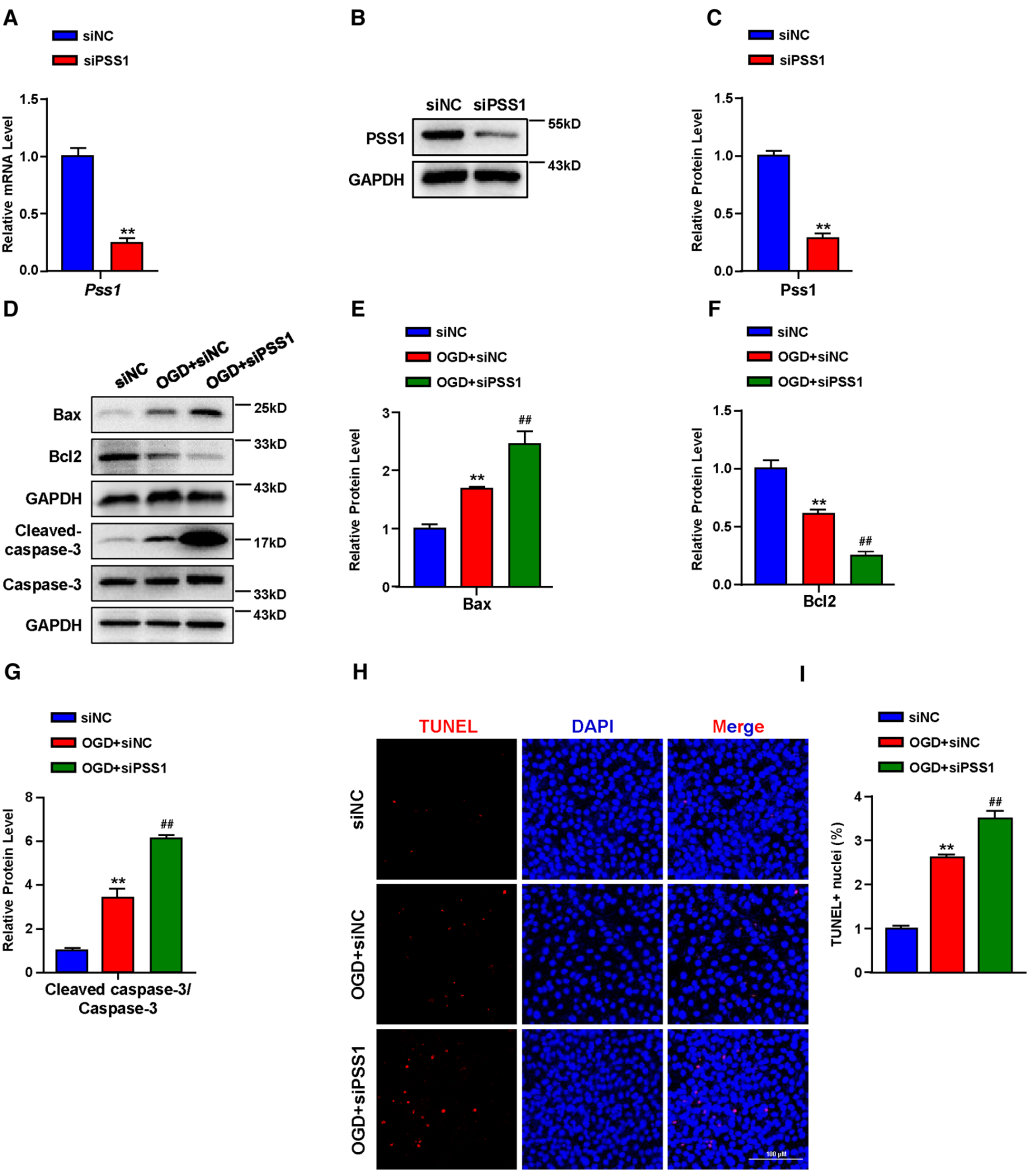
Effects of PSS1 inhibition on cardiomyocyte apoptosis under oxygen-glucose deprivation (OGD). (**A**) mRNA level of *Pss1*. (**B,C**) Western blotting analysis (**B**) and quantification (**C**) of the protein level of PSS1. (**D–G**) Western blotting analysis (**D**) and quantification (**E–G**) of apoptosis-associated protein levels of Bax, Bcl2 and the ratio of cleaved-caspase-3/caspase-3. (**H,I**) Representative images of immunofluorescence staining (**H**) and quantification (**I**) of terminal deoxynucleotidyl transferase dUTP nick end labeling (TUNEL) positive cardiomyocytes. Quantified data are the mean ± SEM of 5 independent experiments. Two-way ANOVA, **P < 0.01 vs. siNC; *^##^P < *0.01 vs. OGD + siNC. Scale bar: 100 µm.

Apoptosis plays a critical role in the pathogenesis of MI. Inhibition of cardiomyocyte apoptosis is an effective means to relieve and prevent myocardial damage caused by MI. To further verify the crucial role of PSS1 in OGD-induced cardiomyocyte apoptosis, we infected cardiomyocytes with adenoviral PSS1 and then treated them with OGD. Western blot analysis of the expression of apoptosis-related proteins demonstrated that overexpression of PSS1 remarkably repressed the apoptotic effects caused by OGD, as evidenced by decreased Bax expression, increased Bcl2 expression and a reduced cleaved-caspase-3/caspase-3 ratio (Figures 5E–H). Meanwhile, TUNEL staining for cellular apoptosis showed that OGD-induced cardiomyocyte apoptosis was largely abrogated by PSS1 overexpression (Figures 5I,J). Conversely, knockdown of PSS1 by siRNA aggravated OGD-induced cardiomyocyte apoptosis, as evidenced by increased Bax expression, decreased Bcl2 expression and an elevated cleaved-caspase-3/caspase-3 ratio compared with OGD group (Figures 6A–G). TUNEL staining displayed silence PSS1 expression aggravated OGD-induced cardiomyocyte apoptosis (Figures 6H,I). Consequently, these findings indicated that PSS1 had an anti-apoptotic effect and played an important protective role in MI injury.

## Discussion

Cardiac repair post-MI is determined by cardiac inflammation and resolution. Growing evidence has revealed that cardiac lipid metabolic perturbations are involved in MI (15, 22–25). However, the metabolic changes in the GPLs profile and the underlying mechanisms involved in MI injury and remodeling remain largely unknown. Our observations suggested that (1) the cardiac GPLs profile was markedly changed in post-MI mice, while the plasma GPLs profile was not altered; (2) the PS family is an important contributor to MI injury; (3) downregulation of PSS1 is responsible for decreased PS synthesis in the cardiac tissue of post-MI mice; (4) cardiomyocytes have high expression of PSS1; and (5) anti-apoptotic effects of PSS1 in cardiomyocytes may play a pivotal role in the protection of MI injury.

GPLs are the main components of the cell membrane based on glycerol. Previous studies have linked GPLs with several disorders, including chronic obstructive pulmonary disease (26), Alzheimer's disease (27), and cancer (28, 29). More attention also has been paid on cardiac GPLs and MI. In terms of cardiac GPLs changes, lysoPC16:0, lysoPC18:0, lysoPC18:2, lysoPC18:1, lysoPC20:4, and lysoPE18:0 in heart tissues were increased by MALDI-MS imaging technology in an experimental MI model (3). In the H9c2 rat cardiomyocyte cell line, multiple lipid variations, including increased lysoPC 18:1, PE34:1, PI38:3, and PC34:1, were observed under ischemic conditions in comparison with controls (4). The present study identified that the cardiac GPLs profile, but not the plasma GPLs profile, was significantly changed in post-MI mice. Similar to these studies, we also observed parallel increases in lysophospholipids in post-MI mouse cardiac tissue, including cardiac lysoPG22:6, lysoPG20:5, lysoPG22:5, lysoPG20:4, lysoPG18:3, lysoPS16:0, lysoPS16:1, lysoPS18:2, lysoPS18:3, lysoPS20:4, lysoPS18:0, lysoPS18:1, lysoPS20:3, lysoPS20:5, and lysoPI14:0, in addition to increased PE34:1, PE32:1, PE32:0, PC30:0, and PG32:0. Nonetheless, the reduced content of PS classes in mouse cardiac tissue is particularly compelling in our model, including cardiac PS40:6, PS38:3, PS40:7, PS38:4, PS38:6, PS40:5, PS38:5, PS36:2, and PS38:2. This discrepancy could partially be due to species-specific differences and technical differences, but the underlying mechanism warrants further investigation.

An early study discovered that patients with ischemic heart disease had high serum levels of anti-PS antibody, suggesting that levels of PS disorder were independent risk factors for ischemic heart disease (30). Subsequent research has shown that PS exerts a protective role in MI repair. David et al. suggested that oral supplementation with PS reduced mouse acute myocardial infarct size and prevented adverse left ventricular remodeling (9). Furthermore, in a rat model of acute MI, targeting PS-presenting liposomes to infarct macrophages promoted angiogenesis and the preservation of small scars and prevented ventricular dilatation and remodeling (10). In the present study, we characterized the significant changes in PS levels involved in post-MI. We found that the levels of PS40:6, PS38:3, PS40:7, PS38:4, PS38:6, PS40:5, PS36:2, PS38:5, and PS38:2 were significantly decreased in cardiac tissues post-MI, and VIP score analysis showed that PS40:6 is the greatest metabolite contributing to MI injury and remodeling. Our data support that disturbance of PS levels plays a prominent role in MI injury and remodeling. Targeted supplementation of PS to heart tissues may be a potential therapeutic approach to promote cardiac repair post-MI.

The balance of PS levels in the body depends on its dynamic synthesis and transformation. Genes involved in these two processes mainly depend on PSS1, PSS2, PEMT and PISD. By analyzing the expression of these genes, we found that the mRNA and protein levels of PSS1, as well as fluorescence staining of PSS1, were markedly reduced in cardiac tissues of post-MI mice. In a previous study, PSS1 deficiency destroyed normal LDL cholesterol transportation and induced toxic cholesterol accumulation by reducing PS levels (13). Similarly, an important finding of the present study is that downregulation of PSS1 in cardiomyocytes may be the leading cause of cardiac PS reduction and MI injury and remodeling. Single cell expression analysis showed that PSS1 is expressed at higher level in cardiomyocytes than other cell types, suggesting a dominant role of cardiomyocyte PSS1 in PS production and MI injury. In particular, the OGD-induced inhibition of PSS1 and the reduction in PS levels were reversed by PSS1 overexpression in cardiomyocytes. Moreover, PSS1 overexpression prevented OGD-induced cardiomyocyte apoptosis, silence PSS1 expression aggravated OGD-induced cardiomyocyte apoptosis. These data indicated a protective role of PSS1 in myocardial damage and injury by reducing cardiomyocyte apoptosis.

In summary, we identified PSS1 as a central regulator of cardiac repair post-MI, and PS levels may be an important diagnostic indicator for MI. Targeting PSS1 in the heart may be an effective approach for treating heart injury after MI.

## Data Availability

The original contributions presented in the study are included in the article, further inquiries can be directed to the corresponding author.
